# Graphical dataset on important medicinal plants used for curing dental issues in Manoor Valley, Mansehra, Pakistan

**DOI:** 10.1016/j.dib.2016.11.025

**Published:** 2016-11-15

**Authors:** Inayat Ur Rahman, Farhana Ijaz, Aftab Afzal, Zafar Iqbal, Niaz Ali, Muhammad Azhar Khan, Muhammad Afzal, Said Muhammad, Ghulam Qadir, Muhammad Asif

**Affiliations:** Department of Botany, Hazara University, Mansehra 21300, KP, Pakistan

**Keywords:** Graphical dataset, Dental issues, Quantitative ethnobotany, Manoor Valley Pakistan

## Abstract

The graphical dataset in this paper is related to the research article entitled “*A novel survey of the ethno medicinal knowledge of dental problems in Manoor Valley (Northern Himalaya), Pakistan”* (I.U. Rahman, F. Ijaz, Z. Iqbal, A. Afzal, N. Ali, M. Afzal, M.A. Khan, S. Muhammad, G. Qadir, M. Asif, 2016) [1]. This article describes how the local community of Manoor Valley practices cultural / traditional knowledge for dental problems. For the recorded data of 25 medicinal plants, six quantitative ethnomedicinal statistical approaches / equations were used. Out of these indices, four were used to measure the most imported and cited medicinal plant species while two for the comparative analysis to evaluate the novelty of work.

**Specifications Table**

**Table:** 

Subject area	*Ethnobotany*
More specific subject area	*Medicinal plants used in curing dental problems*
Type of data	*Figures/ graphs, text file*
How data was acquired	*Survey, interviews, statistical approaches using the indices i.e. use value (UVi), relative frequency citations (RFCs), fidelity level index (FL%), consensus index (CI%), Jaccard similarity index (JI) and Sorensen׳s similarity index (QS).*
Data format	*Raw, analyzed*
Experimental factors	*Questionnaire, statistical indices*
Experimental features	*The relationship between local community and medicinal plants*
Data source location	*Manoor Valley, Mansehra, Pakistan*
Data accessibility	*Data is available with this article*

**Value of the data**

• The relationship between local community and medicinal plants.
• The data presents quantitative ethnobotanical statistical approaches.
• For novelty, ethnomedicinal uses were comparatively analyzed with published literature through similarity indices.

## 1 Data

**Fig. 1:**
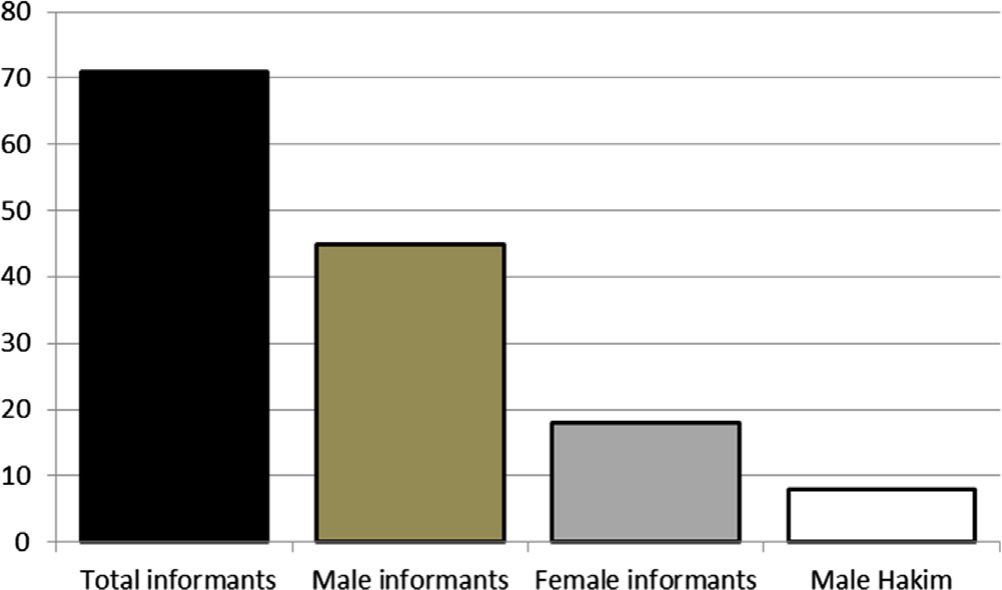
Number of informants interviewed during field work.

The dataset of this article provides information about the medicinal plants used against dental problems in Manoor Valley, Mansehra, Pakistan. Fig. 1 shows the number of informants interviewed, Fig. 3 indicates the growth form of medicinal plants, Fig. 4 reveals the most widely part used. Fig. 5, Fig. 6, Fig. 7 clearly shows the mostly important and widely used medicinal plant species for various dental issues/problems. Fig. 8, Fig. 9 indicates the comparative analysis of reported medicinal plants for current uses with previous documentations.

## 2 Experimental design, materials and methods

**Fig. 2:**
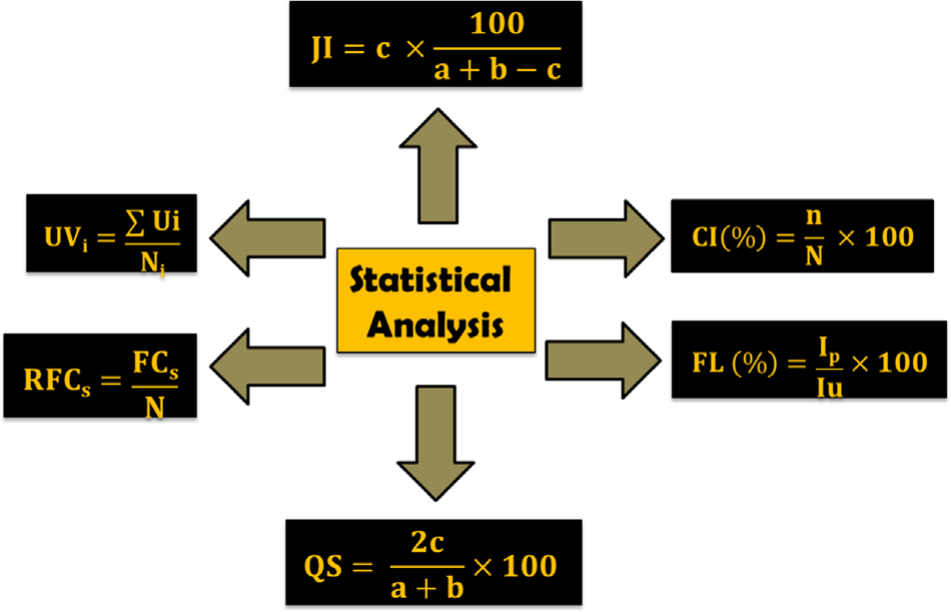
Quantitative ethnomedicinal/statistical indices used to evaluate most cited medicinal plants (UV_i_, RFC_s_, CI and FL) and comparative analysis (JI and QS).

**Fig. 3:**
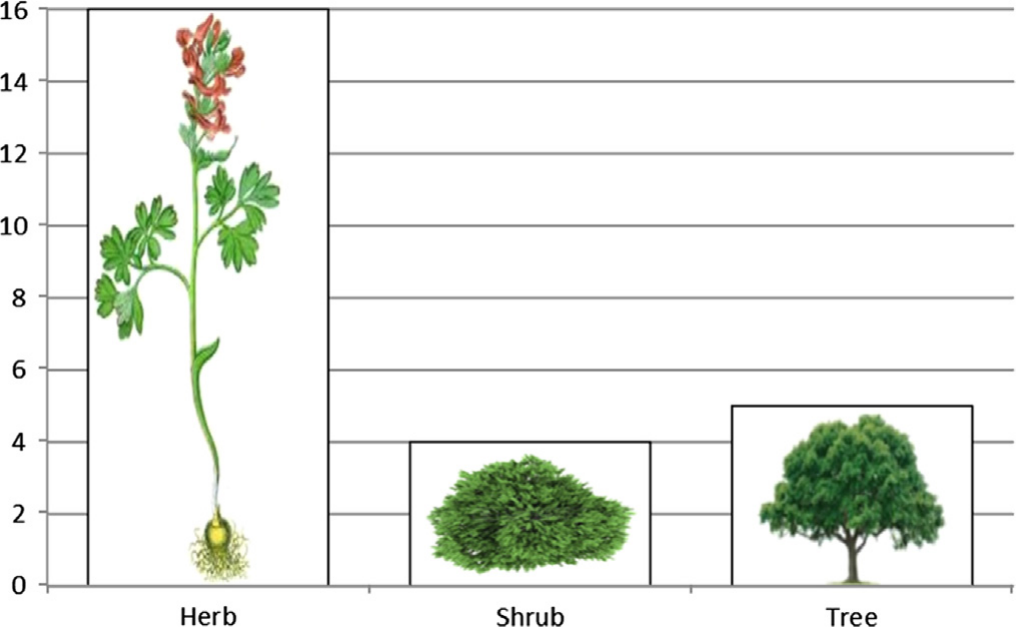
Life form of plants used for ethno medicinal practices.

**Fig. 4:**
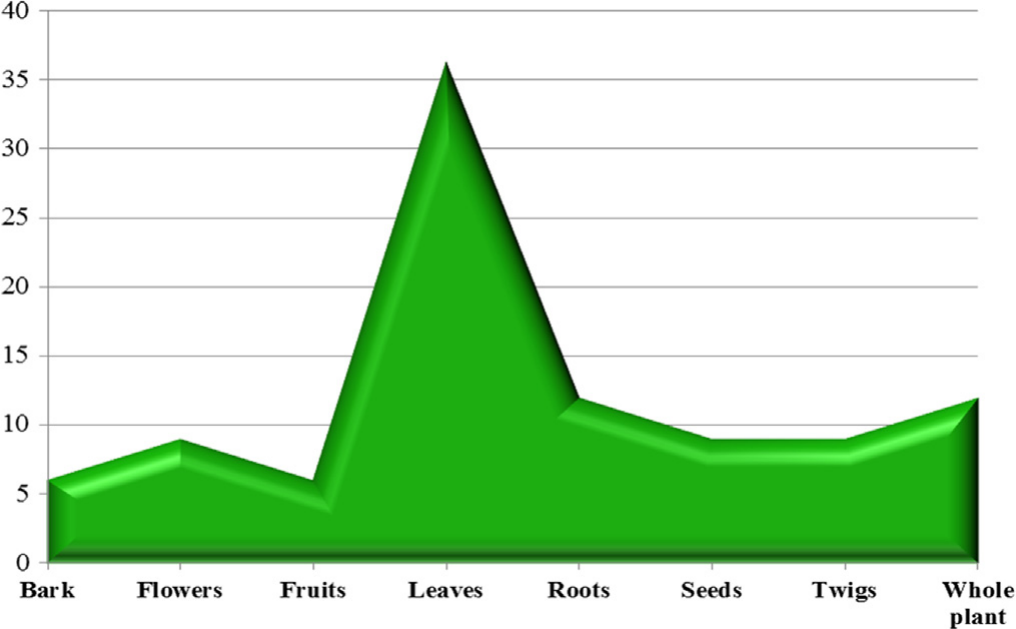
Phyto parts used as traditional medicines for dental problems.

**Fig. 5:**
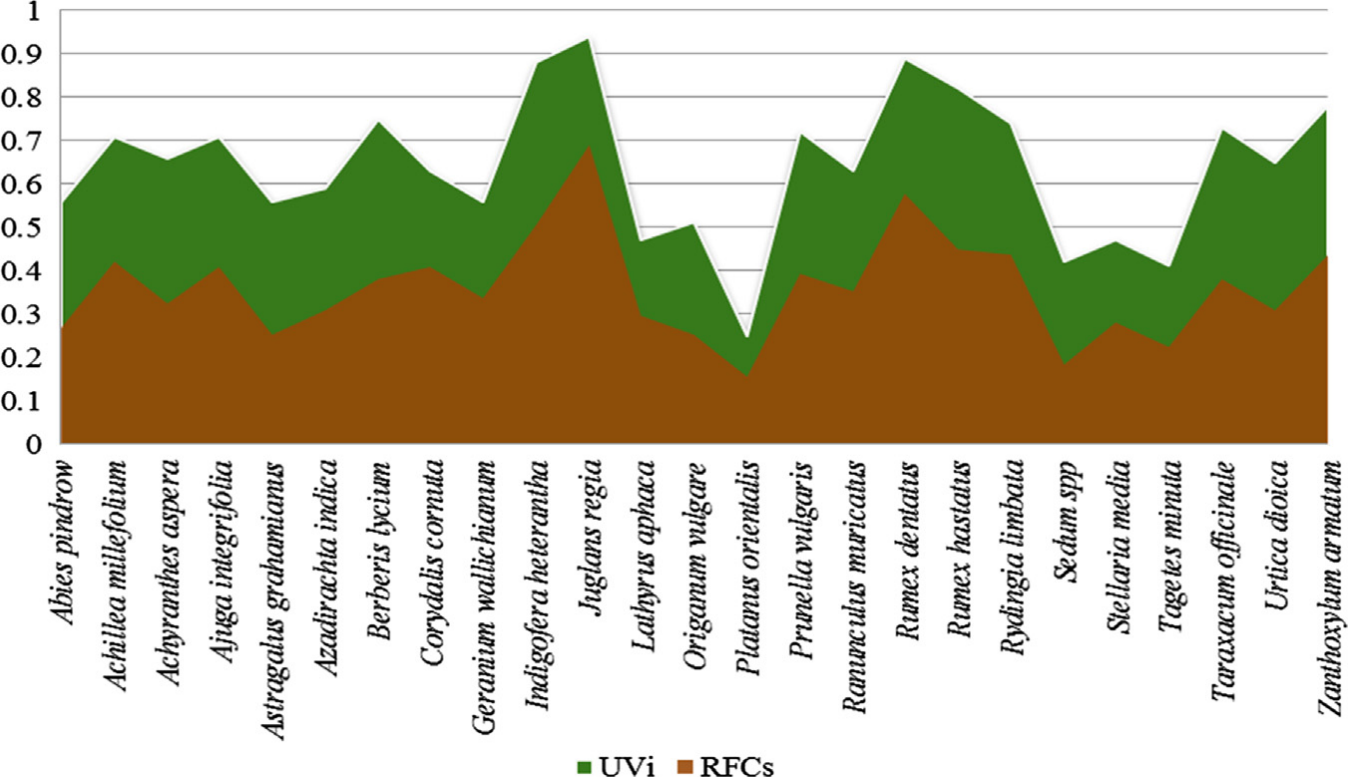
Use value and Relative frequency citations of medicinal plants cited by the informants for various dental problems.

**Fig. 6:**
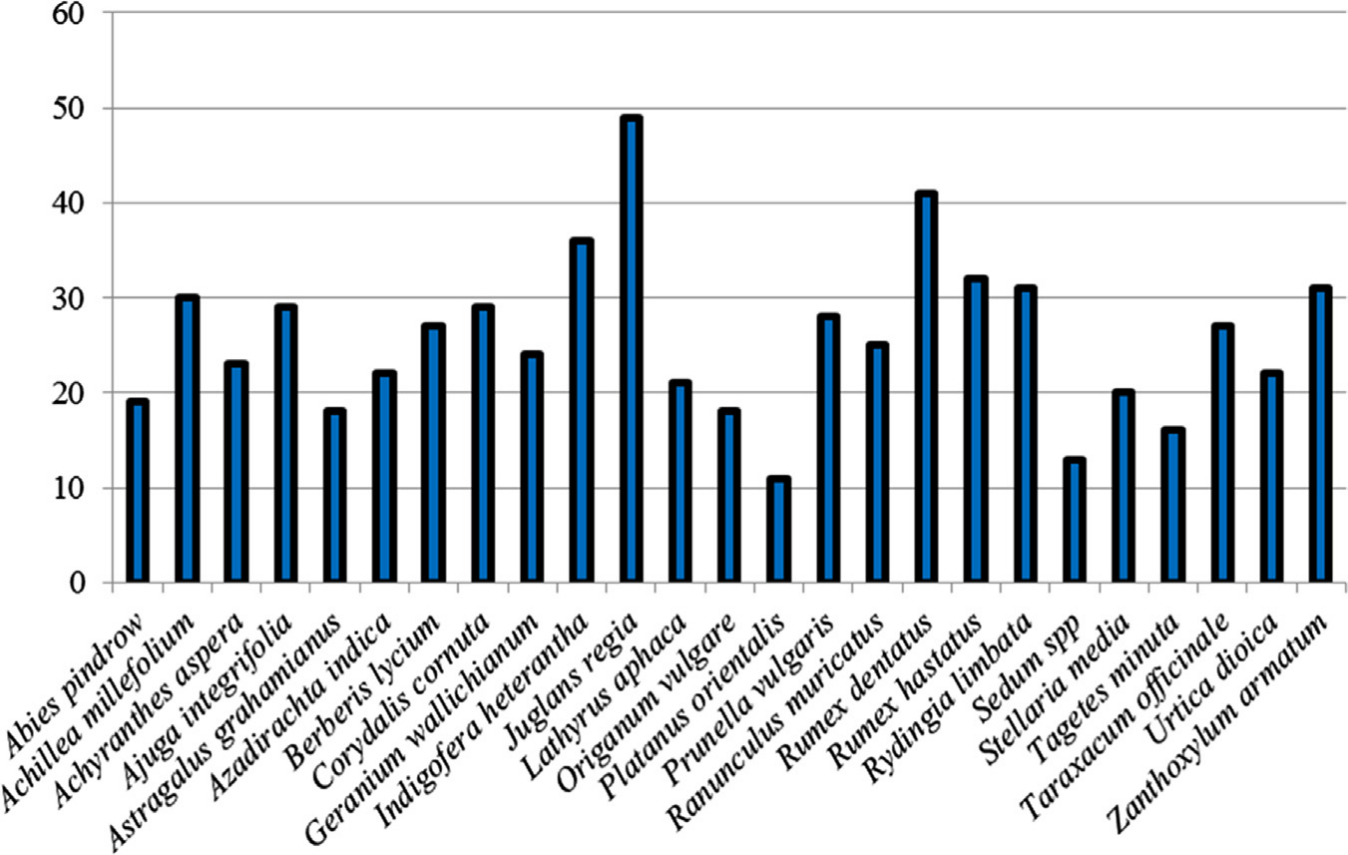
No. of informants cited plant species used for various dental problems.

**Fig. 7:**
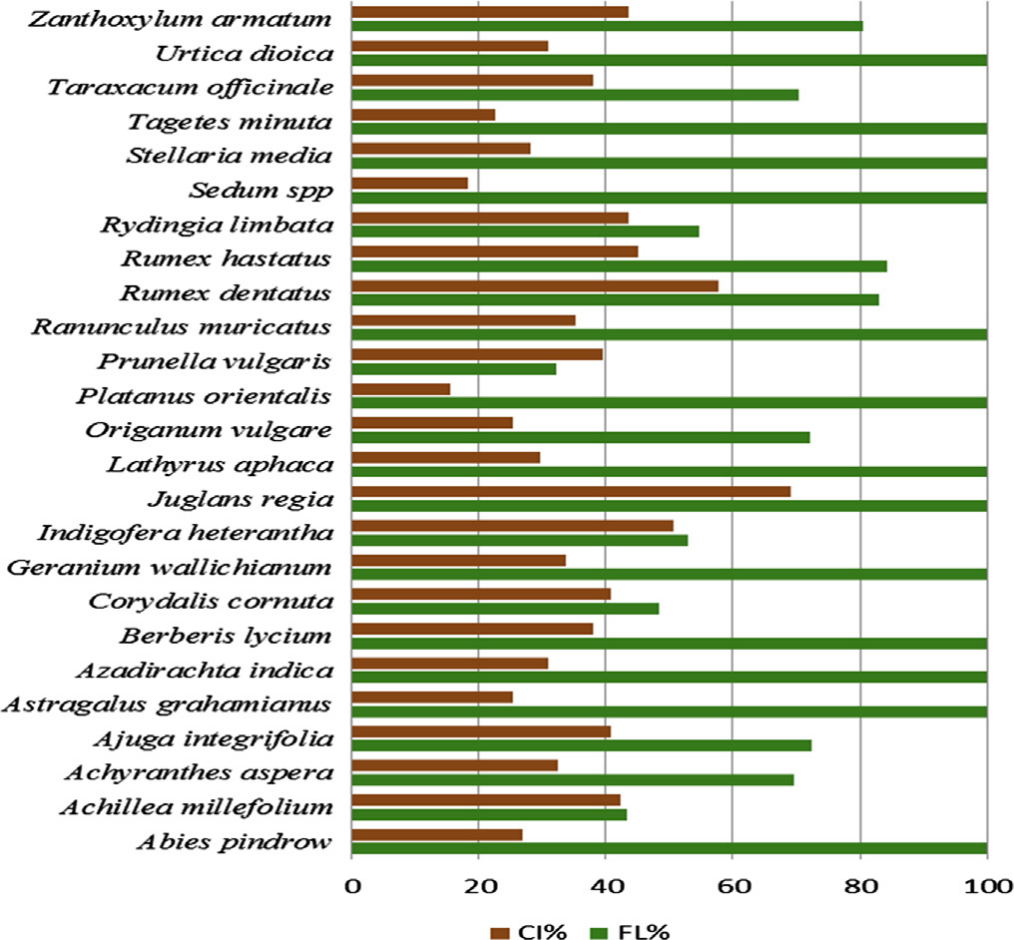
Fidelity Level (%) and Consensus index (%) of medicinal plants cited by the informants for various diseases.

**Fig. 8:**
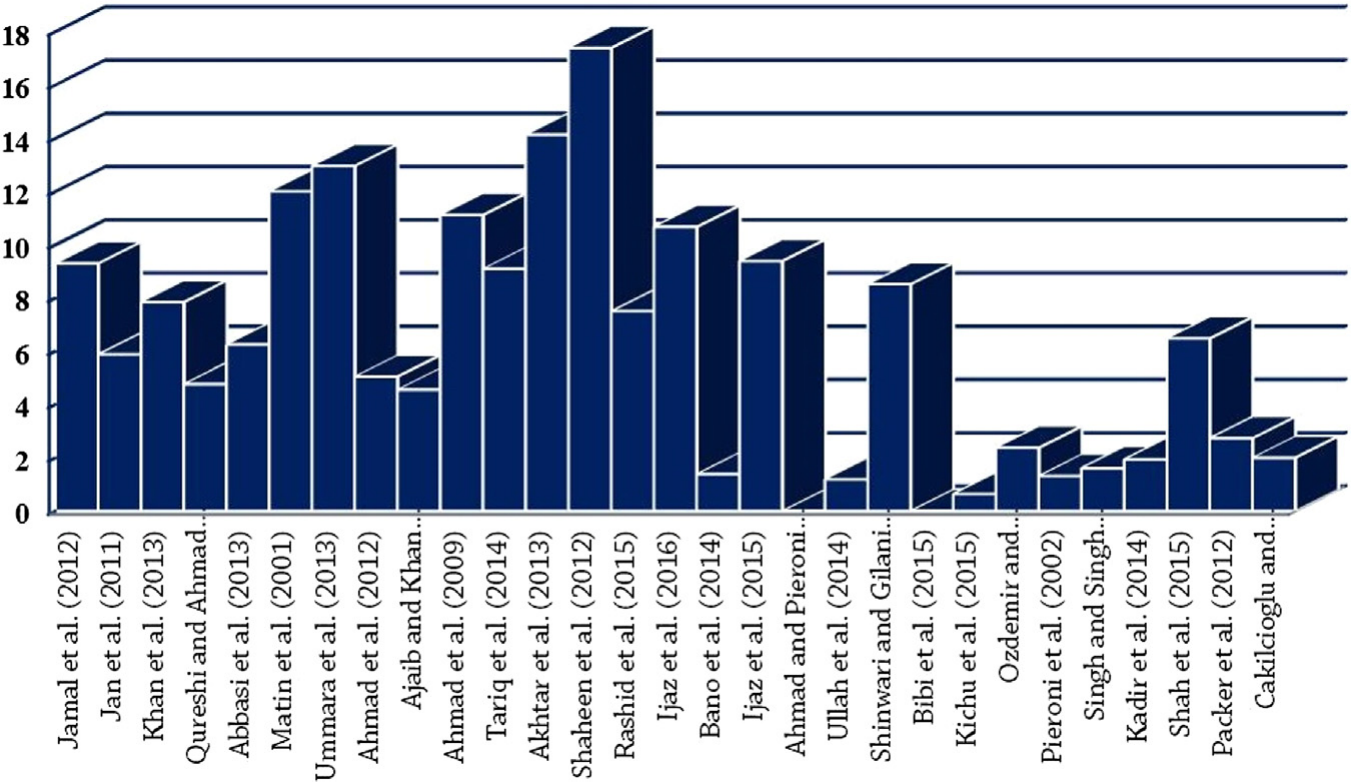
Comparative analysis of the present work [1] with previous studies at regional, national and global level through Jaccard similarity index.

**Fig. 9:**
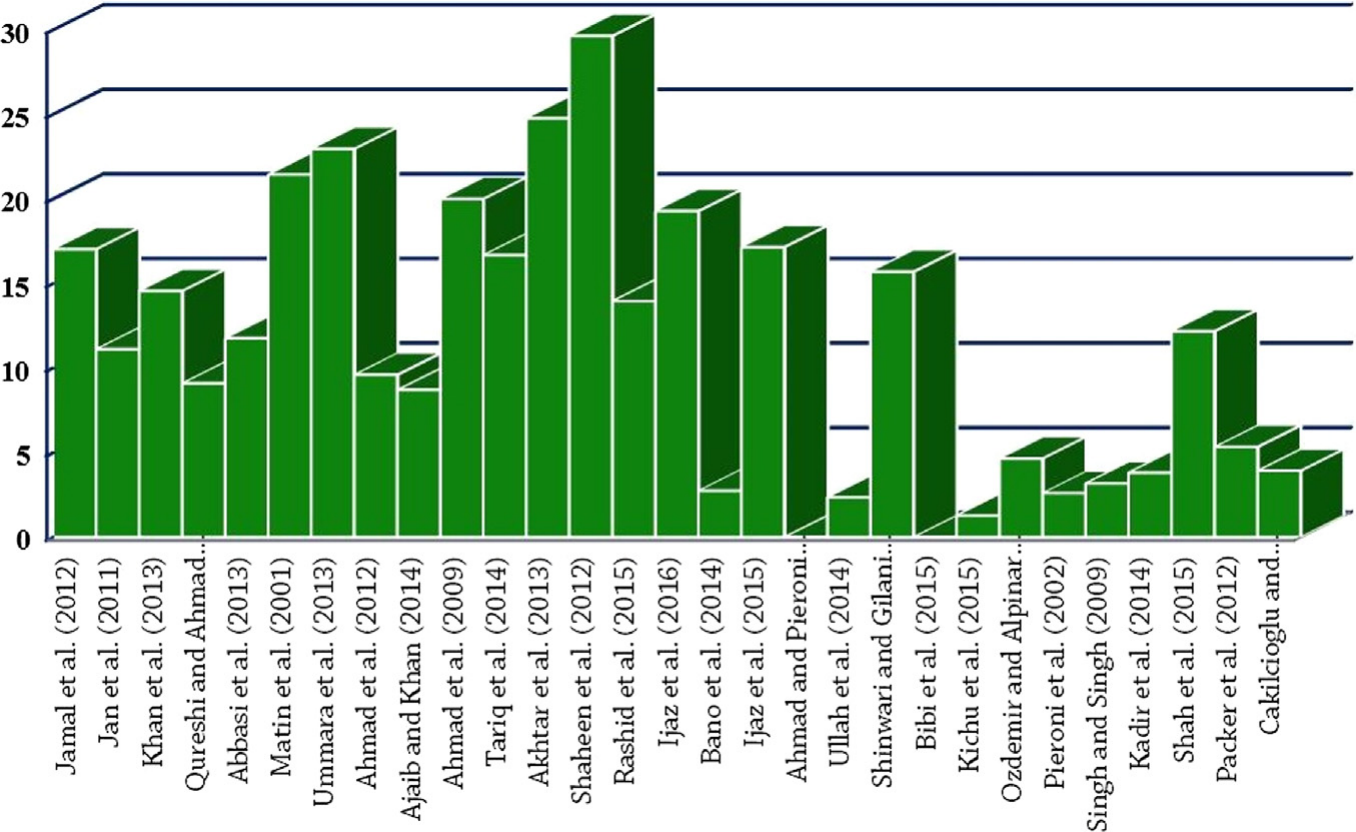
Comparative analysis of the present work [1] with previous studies at regional, national and global level through Sorensen similarity index.

Field surveys were arranged for collection and documentation of medicinal plants of Manoor valley during 2015. Through structured questionnaire, a total of 71 local inhabitants were interviewed randomly. Recorded data were analyzed by using quantitative ethnomedicinal statistical indices/equations (Fig. 2) i.e. use value (UV_i_), relative frequency citations (RFC_s_), fidelity level index (FL%) and consensus index (CI%) [2]. For novel uses all plants were thoroughly checked with previously published articles on the same disorder and analyzed through Jaccard index (JI) and Sorensen׳s similarity index (QS) [3]. Comprehensive discussion and interpretation on these indices and comparative analyses with previous published articles/literature through JI and QS can be found in [1].

## Conflict of Interest

The authors declare that they have no conflict of interests.
